# Hemodynamic analysis for endovascular treatment in small unruptured intracranial aneurysms: a matched comparison study of flow diverter versus LVIS

**DOI:** 10.1186/s41016-021-00266-w

**Published:** 2021-12-01

**Authors:** Jian Liu, Wenqiang Li, Yisen Zhang, Kun Wang, Xinjian Yang, Ying Zhang

**Affiliations:** Department of Interventional Neuroradiology, Beijing Neurosurgical Institute and Beijing Tian Tan Hospital, Capital Medical University, No.119, South 4th Ring West Road, Fengtai District, Beijing, China

**Keywords:** Small intracranial aneurysm, Hemodynamics, Flow diverter, Stent

## Abstract

**Background:**

We compared the treatment of small unruptured intracranial aneurysms (UIAs) with flow diverter and LVIS-assisted coiling to determine the effects of hemodynamic changes caused by different stent and coil packing in endovascular treatment.

**Methods:**

Fifty-one UIAs in 51 patients treated with pipeline embolization device (PED) were included in this study and defined as the PED group. We matched controls 1:1 and enrolled 51 UIAs who were treated with LVIS stent, which were defined as the LVIS group. Computational fluid dynamics were performed to assess hemodynamic alterations between PED and LVIS. Clinical analysis was also performed between these two groups after the match.

**Results:**

There was no difference in procedural complications between the two groups (*P* = 0.558). At the first angiographic follow-up, the complete occlusion rate was significantly higher in the LVIS group compared with that in the PED group (98.0% vs. 82.4%, *P* = 0.027). However, during the further angiographic follow-up, the complete occlusion rate in the PED group achieved 100%, which was higher than that in the LVIS group (98.0%). Compared with the LVIS group after treatment, cases in the PED group showed a higher value of velocity in the aneurysm (0.03 ± 0.09 vs. 0.01 ± 0.01, *P* = 0.037) and WSS on the aneurysm (2.32 ± 5.40 vs. 0.33 ± 0.47, *P* = 0.011). Consequently, the reduction ratios of these two parameters also showed statistical differences. These parameters in the LVIS group showed much higher reduction ratios. However, the reduction ratio of the velocity on the neck plane was comparable between two groups.

**Conclusions:**

Both LVIS and PED were safe and effective for the treatment of small UIAs. However, LVIS-assisted coiling produced greater hemodynamic alterations in the aneurysm sac compared with PED. The hemodynamics in the aneurysm neck may be a key factor for aneurysm outcome.

## Background

Flow diversion is typically reserved for large, giant, or morphologically complex aneurysms [1, 2]. However, large and giant aneurysms represent a small fraction of all cerebral aneurysms, with the majority being small (< 10 mm) in the general population [3]. Recently, the indication of the pipeline embolization device (PED), a type of flow diverter, was expanded to include all wide-neck intracranial aneurysms regardless of their size or location in the internal carotid artery [4]. PED was demonstrated to be safe and effective for the treatment of small aneurysms, with high angiographic occlusion rates [5, 6].

Stent-assisted coiling is also a well-established technique for treatment of unruptured intracranial aneurysms (UIAs), with an excellent efficacy and safety profile. Low-profile visualized intraluminal support (LVIS) is a novel self-expanding nitinol closed-cell braided stent that is widely used in UIAs and was demonstrated to be a feasible, safe, and effective treatment for intracranial aneurysms [7]. The LVIS stent has a smaller cell structure and a higher metal coverage than conventional low metal surface coverage stents (e.g., Neuroform, Enterprise, and Atlas) and is considered to have stronger flow diversion effect than that for conventional stents [8–11]. Thus, the LVIS may further promote complete aneurysm occlusion. However, there is currently no matched comparison study to assess the treatment safety and efficacy between PED and LVIS for use in small UIAs (< 10 mm). More importantly, although many studies suggest that hemodynamics play an important role in aneurysm occlusion and recurrence [12–15], there are limited studies comparing hemodynamics between flow diverter and the LVIS stent for treatment of UIAs.

In the present study, we compared the clinical efficacy of PED and the LVIS stent in matched groups of patients with small UIAs and performed hemodynamic analysis using computational fluid dynamics (CFD).

## Methods

### Patient selection

This was a retrospective, matched, case-control study, which was approved by the ethics committee of our hospital. We reviewed the medical records and image data in our aneurysm database of patients diagnosed with UIAs between August 2014 and December 2019. The patients were selected in accordance with current standards, as follows. The inclusion criteria were (1) small UIAs (< 10 mm) confirmed by CTA, MRA, or DSA; (2) treated with PED or an LVIS stent; (3) had complete clinical records and follow-up data; and (4) had three-dimensional (3D) angiographic images of the UIAs adequate for CFD analysis. Patients with other intracranial tumors, angiostenosis, or angio-malformations (including arteriovenous malformation and cavernous malformation) were excluded from this study.

Angiographic findings were evaluated using the following classification: complete occlusion, residual neck, and residual aneurysm [16, 17]. Aneurysm recurrence was defined as any increase in contrast filling of the aneurysms during follow-up. Two experienced neurointerventionalists (5 years of experience in endovascular treatment) who were independent of the study evaluated the angiography results and radiographic images. Disagreements were resolved by a third neurointerventionalist (10 years of experience in endovascular treatment). A final 51 UIAs in 51 patients treated with PED were included in this study (PED group).

Matched control cases were selected by a retrospective review of all small UIAs with LVIS stent placement seen in our clinic during the specified timeframe. We matched controls 1:1 and enrolled 51 UIAs treated with the LVIS stent (LVIS group). The primary basis for the matched controls was the treatment method and aneurysm size. Additional matching was performed in the following order: demographic factors (age and gender), the location, and wide-neck aneurysms. For all included cases, clinical data (age, gender, presentation, smoking, hypertension, diabetes mellitus, hyperlipidemia, and pre-procedure mRS), morphologic data (aneurysm size, wide neck, multiple aneurysms, fusiform-dissecting aneurysm, and aneurysm location), and procedural and follow-up data (treatment strategy, multiple stents, technical success rate, procedural complications, angiographic results and duration at the first and last follow-up, recurrence, and mRS at last follow-up) were collected from medical records and imaging studies.

### Interventional procedures

All patients were treated with standard dual-antiplatelet therapy (100 mg aspirin and 75 mg clopidogrel) at least 5 days before the procedure. The platelet function test (thromboelastography and genotype of CYP2C19) was performed to identify the hyporesponders, in whom the antiplatelet regimen was further adjusted. All procedures were performed under general anesthesia, and full procedural heparinization was used to achieve a targeted activated clotting time of 250–300 s. The jailing technique was the typical method used for stent-assisted coiling, in which a stent was deployed after the microcatheter was in position. Following the procedure, dual antiplatelet therapy was continued for at least 6 weeks, and aspirin was continued for 6 months thereafter.

For patients treated with PED, we used a triaxial supporting system to access the aneurysm. The PED was introduced via the Marksman microcatheter, delivered to the parent artery, and then deployed. Several endovascular techniques (included use of wires, catheters, or balloon angioplasty) were used if the device was inadequately expanded. Following stent delivery, control angiography was performed in the working angles for treatment, as well as anterior-posterior and lateral angiography. After the procedure, dual antiplatelet therapy was continued for 3–6 months, followed by indefinite aspirin monotherapy.

### Vascular modeling, CFD simulation, and hemodynamic analysis

Patient-specific aneurysm morphologies were reconstructed and obtained from 3D rotational angiography images. The 3D geometry surface was displayed, segmented, and smoothed using software (Geomagic Studio v12.0; Geomagic, Research Triangle Park, NC, USA), and the geometries were saved in standard tessellation language format. We simulated the hemodynamics of the aneurysms using previously developed computational modeling methods [15]. A novel virtual stenting technique [9] and porous media method [18] were used to simulate the in vivo stent and coil mass in the aneurysm dome region. Finite-volume elements for CFD simulation were created by merging the virtual stent with the aneurysm geometry using mesh generation software (ICEM CFD v14.0; ANSYS Inc., Canonsburg, PA, USA) to generate > 1 million finite-volume tetrahedral elements. The largest element size was set at 0.2 mm, and the element size on the stent was set to 1/3 of the width of the stent strut.

The flow-governing Navier-Stokes equations were solved using software (ANSYS CFX v14.0; ANSYS Inc.). Some assumptions were applied, including laminar, incompressible, and Newtonian blood flow and a rigid vessel wall with no-slip boundary conditions. The blood density and dynamic viscosity were specified as 1060 kg/m^3^ and 0.004 Pa/s, respectively. A representative pulsatile period velocity profile was obtained using transcranial Doppler imaging and set as the inflow boundary condition. The outlet pressure conditions at outlet arteries in our study were imposed to 0 Pa. The flow waveforms were scaled to achieve a mean inlet wall shear stress of 15 dyne/cm under pulsatile conditions. Three cardiac cycle simulations were performed to establish numeric stability. To confirm stability, the results from the third cardiac cycle were collected as the output for the final analyses.

Next, we post-processed and visualized the results of these simulations using the ANSYS CFD-Post software. The hemodynamic results at peak systole were carefully examined. The average flow velocity at the aneurysm neck plane was calculated and the aneurysm neck plane was created at the location where the aneurysm sac pouched outward from the parent artery. The average flow velocity inside the aneurysm and the mean wall shear stress (WSS) on the whole aneurysm wall were also calculated. We defined the reduction ratios of the parameter as (pretreatment parameter − post-treatment parameter)/pretreatment parameter. For hemodynamic parameters, the reduction ratios of the flow velocity at the aneurysm neck plane, the flow velocity in the aneurysm, and the WSS on the aneurysm were analyzed.

### Statistical analysis

We performed a matched case-control analysis using conditional logistic regression. Data are presented as mean ± standard deviation or median (quartile) for quantitative variables and as frequency for qualitative variables. For qualitative data, the *χ*^2^ test or the Fisher’s exact test was used to compare the differences between the PED group and the LVIS group. For quantitative data, the Mann-Whitney *U* test was used to compare the two groups. A *P* value < 0.05 was considered statistically significant. Statistical analyses were performed using statistical software (IBM SPSS Statistics for Windows v21.0; IBM Corp., Armonk, NY, USA).

## Results

### Patient and aneurysm characteristics

After matching, 51 UIAs with PED (PED group) and 51 UIAs with LVIS (LVIS group) were enrolled in this study. All covariates were statistically indistinguishable between the two groups after matching based on similarities in patient and aneurysm characteristics (Table 1).

**Table 1:** Patient and aneurysm characteristics

	PED group (*n* = 51)	LVIS group (*n* = 51)	*P* value
Age	52.90 ± 8.03	52.35 ± 7.95	0.729
Female, %	12 (23.5)	12 (23.5)	1.000
Presentation			0.129
**Asymptomatic**, %	3 (5.9)	9 (17.6)	
**Headache**, %	34 (66.7)	33 (64.7)	
**Neurological deficits**, %	12 (27.5)	9 (17.6)	
**Smoking**, %	4 (7.8)	5 (9.8)	0.727
**HTN**, %	16 (31.4)	21 (41.2)	0.303
**DM**, %	4 (7.8)	2 (3.9)	0.400
**HLD**, %	6 (11.8)	4 (7.8)	0.505
**Pre-procedure mRS**	**1.22 ± 0.54**	**1.00 ± 0.60**	**0.059**
Aneurysm size	5.22 ± 2.45	4.86 ± 1.66	0.384
Wide-neck aneurysm, %	48 (94.1)	47 (92.2)	0.695
Multiple aneurysms, %	13 (25.5)	9 (17.6)	0.336
**Fusiform-dissecting**, %	6 (11.8)	3 (5.9)	0.295
**Aneurysm location**			0.141
**Anterior**, %	45 (88.2)	49 (96.1)	
**Posterior**, %	6 (7.8)	2 (3.9)	

Neurological deficits included oculomotor paralysis and weakness of limbs; *HTN* hypertension; *DM* diabetes mellitus; *HLD* hyperlipidemia

### Aneurysm treatment, complications, and angiographic and clinical outcomes

In the PED group, 34 patients (66.7%) were treated with PED alone and 17 patients (33.3%) were treated with a combination of PED and coils. All cases in the LVIS group were treated with stent-assisted coiling. Procedural complications occurred in 2 patients (3.9%) in the PED group versus 1 patient (2.0%) in the LVIS group (*P* = 0.558). No procedure-related death or intracranial hemorrhage was reported during the procedure or follow-up in either group. At the first angiographic follow-up, the complete occlusion rate was significantly higher in the LVIS group compared with that in the PED group (98.0% vs. 82.4%, respectively; *P* = 0.027). However, at further angiographic follow-up, the complete occlusion rate was 100% in the PED group, which was higher than that in the LVIS group (98.0%; Table 2). One case in the LVIS group showed recurrence at 1-year follow-up.

**Table 2:** Treatment, complications, angiographic, and follow-up results

	PED group (*n* = 51)	LVIS group (*n* = 51)	*P* value
**Treatment strategy**			< 0.001*
**Stent alone**, %	34 (66.7)	0 (0.0)	
**Stent + coiling**, %	17 (33.3)	51 (100.0)	
**Technical success rate, %**	50 (98.0)	50 (98.0)	**1.000**
**Multiple stents**	3 (5.9)	2 (3.9)	0.647
**Procedure complications, %**	**2 (3.9)**	**1 (2.0)**	**0.558**
**First follow-up duration, mo**	6.33 ± 1.84	6.94 ± 1.50	0.071
**Angiographic results at first follow-up**			0.027*
**Complete occlusion**, %	42 (82.4)	50 (98.0)	
**Neck remnant**, %	2 (3.9)	0 (0.0)	
**Sac remnant**, %	7 (13.7)	1 (2.0)	
**Last follow-up duration, mo**	11.7 ± 4.98	**12.47** ± 6.91	0.533
**Angiographic results at last follow-up**			0.315
**Complete occlusion**, %	**51 (100.0)**	50 (98.0)	
**Recurrence, %**	**0 (0.0)**	1 (2.0)	
**Retreatment, %**	**0 (0.0)**	1 (2.0)	0.315
**Last follow-up mRS**	**0.16** ± 0.54	0.14 ± 0.40	0.836

*Mo* months; *mRS* modified Rankin score

### Hemodynamic comparisons between the PED group and the LVIS group

**Fig. 1:**
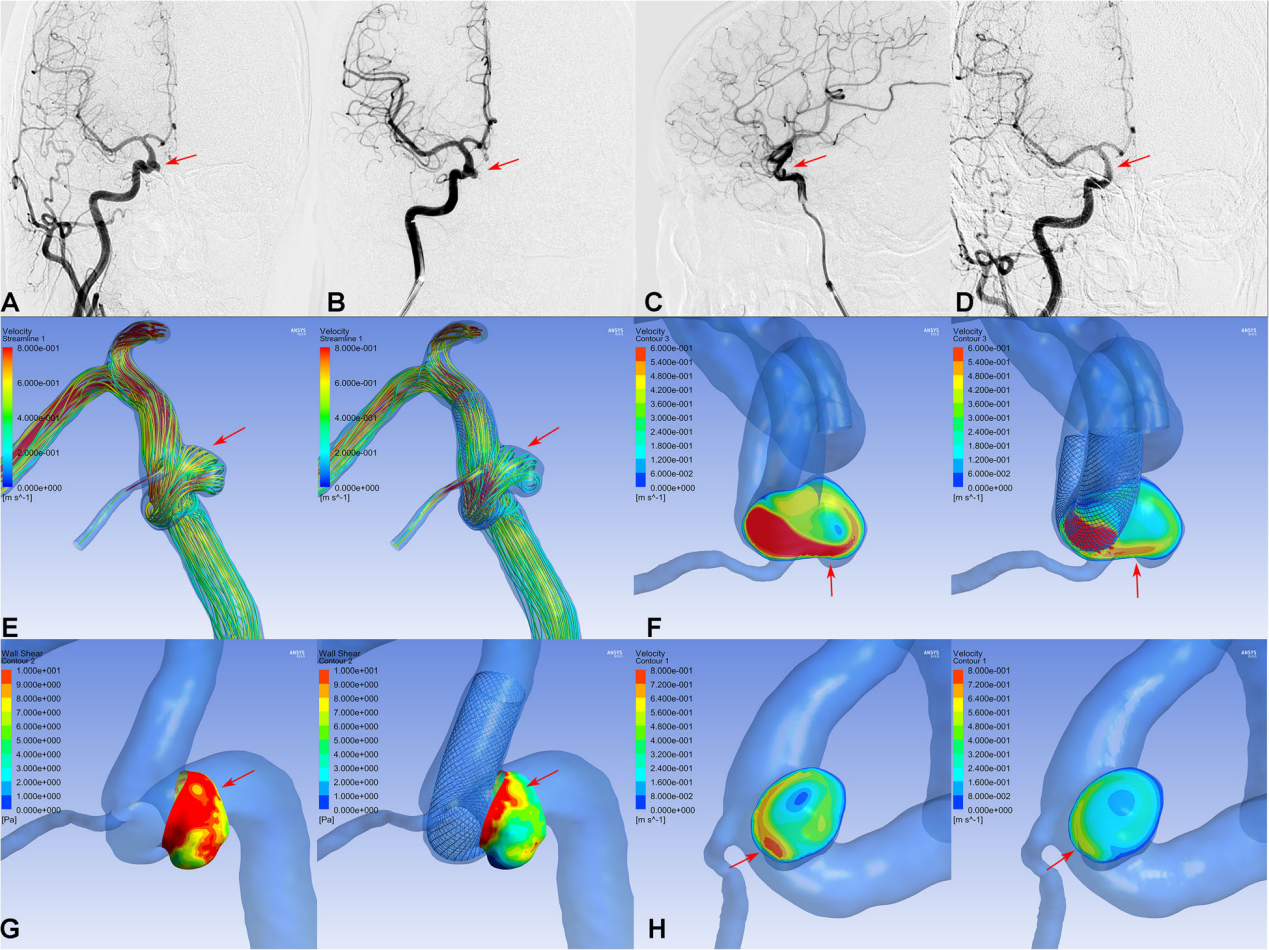
Digital subtraction angiography (DSA) showed a small aneurysm treated with pipeline alone (**A**, **B**, **C**, **D** arrows). Compared with the preprocedural angiographic image (**A**), the aneurysm showed intraaneurysmal contrast stasis in the immediately postprocedural angiographic image (**B**, **C** arrow). At 6-month follow-up, DSA indicated that the aneurysm was occluded completely (**D**, arrow). In hemodynamic simulation, compared with preprocedural results (left **E**, **F**, **G**, **H**), the velocity and WSS of the aneurysm had decreased after treatment (right **E**, **F**, **G**, **H**). However, the blood flow velocity near the aneurysmal neck remained concentrated (**F**, arrows), the wall shear stress of the region near the aneurysmal neck remained high (**G**, arrows). The velocity of the aneurysm neck plane was markedly reduced (**H**, arrows)

**Fig. 2:**
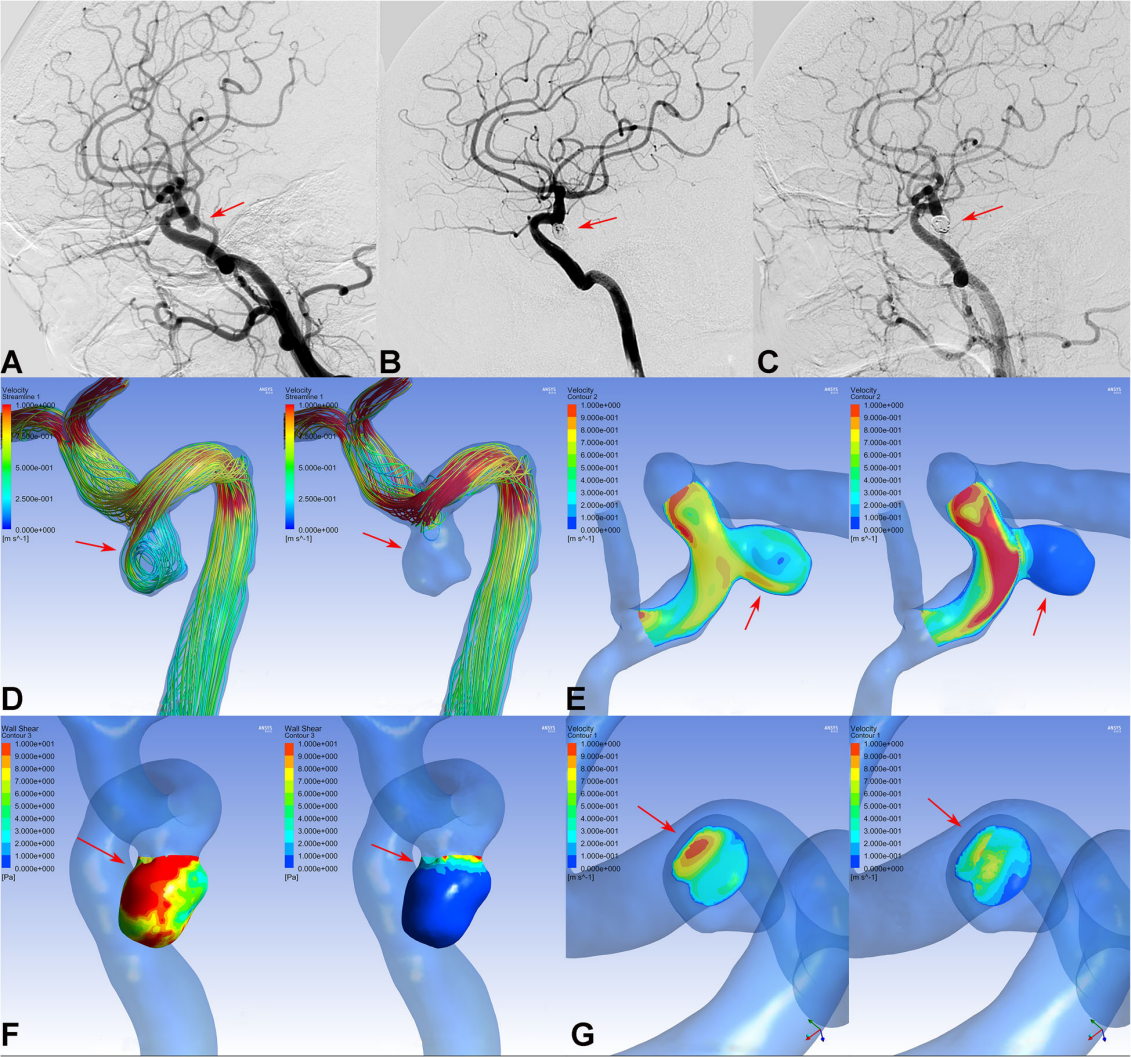
DSA showed a complete occluded small aneurysm treated by LVIS stent-assisted coiling (**A**, **B**, **C**, arrows). In immediately postprocedural angiographic images, the aneurysm showed a residual neck (**B**, arrow). At 6-month follow-up, DSA indicated that the aneurysm had complete occlusion (**C**, arrow). In hemodynamic simulation, the velocity streamline was decreased (**D**, arrows), velocity in the aneurysm and wall shear stress was decreased (**E** and **F**, arrows), and the velocity of the aneurysm neck plane was also markedly reduced (**G**, arrows) after treatment

There were no significant differences in hemodynamic parameters (velocity on the neck plane, velocity in the aneurysm, or WSS on the aneurysm) before treatment between the PED group and the LVIS group (Table 3). However, after treatment, the PED group showed a significantly higher velocity in the aneurysm (0.03 ± 0.09 vs. 0.01 ± 0.01, respectively; *P* = 0.037) and WSS on the aneurysm (2.32 ± 5.40 vs. 0.33 ± 0.47, respectively; *P* = 0.011) compared with the LVIS group. Consequently, the reduction ratios of velocity in the aneurysm and WSS on the aneurysm after treatment were also markedly higher in the LVIS group compared with those in the PED group (*P* < 0.001 for both parameters, Figs. 1 and 2). However, there were no differences in the reduction ratio of the velocity on the neck plane after treatment between the two groups. Considering the coil usage in both groups, the coils showed significant flow remodeling effect on the aneurysm sac. Given that there were no differences in recurrence between the two groups, flow velocity on the aneurysm neck may be a key factor associated with recurrence.

**Table 3:** Hemodynamic parameter comparisons between the two groups

	PED group (*n* = 51)	LVIS group (*n* = 51)	*P* value
**Velocity on the neck plane**			
Pre-operation, m/s	0.23 ± 0.14	0.26 ± 0.13	0.317
Post-operation, m/s	0.11 ± 0.06	0.12 ± 0.10	0.600
Reduction ratio (%)	49.34 ± 18.96	55.73 ± 23.28	0.132
**Velocity in the aneurysm**			
Pre-operation, m/s	0.10 ± 0.12	0.10 ± 0.09	0.853
Post-operation, m/s	0.03 ± 0.09	0.01 ± 0.01	0.037*
Reduction ratio (%)	70.69 ± 32.32	91.53 ± 8.28	< 0.001*
**WSS on the aneurysm**			
Pre-operation, Pa	3.58 ± 3.84	3.56 ± 2.43	0.983
Post-operation, Pa	2.32 ± 5.40	0.33 ± 0.47	0.011*
Reduction ratio (%)	45.55 ± 44.31	91.20 ± 9.82	< 0.001*

*WSS* wall shear stress

### Hemodynamic comparisons among the PED alone cases, PED with coils cases, and LVIS cases

As shown in Table 4, we further compared the cases with coils between the two groups (PED with coils cases versus LVIS cases) in hemodynamics and also compared the PED combined coil cases with PED alone cases.

**Table 4:** Hemodynamic parameter comparisons: PED alone versus PED with coils and PED with coils versus LVIS with coils

	PED alone (*n* = 34)	PED with coils (*n* = 17)	*P* value	PED with coils (*n* = 17)	LVIS with coils (*n* = 51)	*P* value
**Velocity on the neck plane**						
Pre-operation, m/s	0.23 ± 0.13	0.23 ± 0.16	0.542	0.23 ± 0.16	0.26 ± 0.13	0.172
Post-operation, m/s	0.11 ± 0.06	0.11 ± 0.06	0.865	0.11 ± 0.06	0.12 ± 0.10	0.772
Reduction ratio (%)	50.50 ± 17.57	47.03 ± 21.86	0.313	47.03 ± 21.86	55.73 ± 23.28	0.071
**Velocity in the aneurysm**						
Pre-operation, m/s	0.11 ± 0.14	0.07 ± 0.07	0.675	0.07 ± 0.07	0.10 ± 0.09	0.226
Post-operation, m/s	0.04 ± 0.10	0.02 ± 0.02	0.689	0.02 ± 0.02	0.01 ± 0.01	0.069
Reduction ratio (%)	66.75 ± 37.39	78.55 ± 16.81	0.436	78.55 ± 16.81	91.53 ± 8.28	< 0.001*
**WSS on the aneurysm**						
Pre-operation, Pa	3.99 ± 4.39	2.76 ± 2.28	0.617	2.76 ± 2.28	3.56 ± 2.43	0.172
Post-operation, Pa	2.73 ± 6.50	1.50 ± 1.65	0.719	1.50 ± 1.65	0.33 ± 0.47	< 0.001*
Reduction ratio (%)	41.24 ± 50.30	54.17 ± 28.29	0.719	54.17 ± 28.29	91.20 ± 9.82	< 0.001*

*WSS* wall shear stress

Compared the coiled cases, the results between PED (17 cases) and LVIS (51 cases) were similar as the results from the comparison between the PED group (51 cases) and LVIS group (51 cases). The velocity and WSS in the aneurysm in the LVIS group showed much higher reduction ratios. In the PED group, the coil mass created more hemodynamic reductions in the aneurysm sac. However, there were no significantly hemodynamic differences between PED alone cases and PED with coils cases.

## Discussion

In the present study, we found that PED had a comparable efficacy and safety profile to LVIS stents in similar patient populations with small UIAs. However, although there was a similar complete occlusion rate between the two groups at the last follow-up, the LVIS stent had a higher complete occlusion rate at early follow-up. Furthermore, based on hemodynamic findings, LVIS-assisted coiling showed a higher flow diversion effect than that for PED.

### Angiographic and clinical outcomes in small UIAs with PED and LVIS treatment

As the initial indications for PED include large or giant aneurysms, few studies have reported the use of PED in small aneurysms. Furthermore, despite reports showing a satisfactory efficacy and safety of PED, as yet, there are no direct matched comparisons with stent-assisted coiling in small UIAs, making it difficult to determine the optimal treatment strategy for these lesions.

In a study comparing the efficacy and safety of flow diversion versus coiling treatment in small UIAs, both treatments were found to provide high occlusion rates, low retreatment rates, and additional morbidity in small, simple aneurysms [6]. However, the LVIS stent was not examined in that study. In another study of patients with middle cerebral arterial aneurysms (144 aneurysms in total), Lv et al. [19] reported that both the LVIS stent and PED treatment were effective and safe, with a good prognosis rate and low recurrence. However, that study included 25 large aneurysms and 25 ruptured aneurysms. Furthermore, Lu et al. [20] reported that LVIS stents and PED had acceptable rates of complete occlusion for UIAs, while peri-operative complications were more frequent in the PED group, and large aneurysm size (> 10 mm) was associated with recanalization. Finally, in a meta-analysis of 1451 patients with 1654 aneurysms treated with flow diversion, the rates of postoperative subarachnoid hemorrhage, ICH, and ischemic stroke rate were 3%, 3%, and 6%, respectively, while patients with small aneurysms had a significantly lower rate of postoperative subarachnoid hemorrhage and ischemic stroke than those with larger aneurysms [21]. Thus, the authors concluded that flow diversion in small aneurysms had a superior safety to that in large aneurysms. Considering the risk of post-operative hemorrhage event and the high metal coverage of the device, the use of PED for aneurysms that can be effectively treated with other devices should be cautioned. In the present study, although the short-term follow-up was better in the LVIS group, both PED and LVIS were safe and effective for management of small UIAs at longer follow-up.

### Hemodynamic differences between PED and LVIS treatment

Hemodynamics play an important role in aneurysm outcome after embolization treatment [11–13, 15]. Stent-assisted coiling was developed from the concept that scaffolding in the parent artery of wide-necked aneurysms prevents coil herniation (by stabilizing the coil) and aneurysm recurrence. The stent may also lead to progressive endothelialization and reconstruction of the parent vessel, while numerous studies have shown that stents produce flow-remodeling effects to decrease aneurysm hemodynamics. Furthermore, flow diverters are designed to remodel the flow impact and reconstruct the parent vessel as the high metal coverage [22–24]. Wang et al. [9] reported that LVIS stents exert hemodynamic effects on cerebral aneurysms and that a single LVIS stent caused more flow reductions than two Enterprise stents, but less than the PED. However, that was a single case analysis study with no association with clinical outcomes. We also recently compared the hemodynamic effects of LVIS, compacted LVIS, and PED and found that compacted LVIS had a comparable flow diverting effect to PED [25].

Despite these studies examining the hemodynamic actions of LVIS, the effects on aneurysm outcome remain unclear. In a retrospective analysis, Zhang et al. [15] reported that a significant reduction in flow velocity at the neck plane was the most important factor for prevent aneurysms recanalization, although there were no PED cases. In a study examining the risk factors for aneurysm recurrence in 52 primary-coiling aneurysm cases after simulation, Damiano et al. [26] also reported that larger aneurysm size and neck, less coil packing, and higher intra-aneurysmal flow before and after coiling predicted recurrence. However, that study had a small sample size and no stent or flow diverter cases were included. Based on the encouraging results for treatment of large aneurysms using flow diverters with or without loose packing coils, we considered that hemodynamics may be the most important predictor of aneurysm outcome. In the present study, the reduction ratios after treatment were approximately 50% in both groups, while the aneurysm outcomes at last follow-up were satisfactory in both groups, as previously reported. Additionally, the LVIS with coils showed a significant stronger flow remodeling effect in the aneurysm sac than for PED, which is likely because of the dense packing of coils achieved when using LVIS. By contrast, loose packing is preferred when using PED. We also showed the similar results between PED combined coil cases (17 cases) and LVIS cases in hemodynamics. Finally, the key hemodynamic factors associated with aneurysm outcome included flow velocity on the neck, rather than hemodynamics in the aneurysm sac, as we previously reported [11, 15, 27].

There are some limitations to the present study. First, the small sample size may have influenced our results. Thus, the clinical safety and efficacy should be confirmed in larger studies. Furthermore, although we used two follow-up times, the follow-up interval was short. Next, the various assumptions used for the vessel and aneurysm blood flow simulations (e.g., a rigid wall, laminar flow, and Newtonian blood) may have affected the hemodynamic results. Finally, the mechanisms of aneurysm recurrence cannot be explained by hemodynamics alone. Thus, a comprehensive study examining multiple factors is still required.

## Conclusions

Both LVIS and PED were safe and effective for the treatment of small UIAs. However, LVIS-assisted coiling produced greater hemodynamic alterations in the aneurysm sac compared with PED. The hemodynamics in the aneurysm neck may be a key factor for aneurysm outcome.

## Data Availability

All study data are stored by information system at Department of Interventional Neuroradiology, Beijing Neurosurgical Institute and Beijing Tian Tan Hospital, Capital Medical University. All the data are available after contacting the corresponding author.

## Abbreviations

PED: Pipeline embolization device
UIAs: Unruptured intracranial aneurysms
LVIS: Low-profile visualized intraluminal support
CFD: Computational fluid dynamics
3D: Three-dimensional
WSS: Wall shear stress
